# Supplementary material for: Two Antagonistic Microtubule Targeting Drugs Act Synergistically to Kill Cancer Cells
Source: Cancers (Basel). 2020 Aug 6;12(8):2196. doi: 10.3390/cancers12082196 (PMC7463452; doi:10.3390/cancers12082196)
Supplement: Supplementary file 1 [file cancers-12-02196-s001.zip › cancers-827652 - final - supplementary material/cancers-827652 - supplementary.pdf]

Supplementary material

Two Antagonistic Microtubule Targeting Drugs Act Synergistically to Kill Cancer Cells

Lauralie Peronne, Eric Denarier, Ankit Rai, Renaud Prudent, Audrey Vernet, Peggy Suzanne, Sacnicté Ramirez-Rios, Sophie Michallet, Mélanie Guidetti, Julien Vollaire, Daniel Lucena-Agell, Anne-Sophie Ribba, Véronique Josserand, Jean-Luc Coll, Patrick Dallemagne, J. Fernando Díaz, María Ángela Oliva, Karin Sadoul, Anna Akhmanova, Annie Andrieux and Laurence Lafanechère

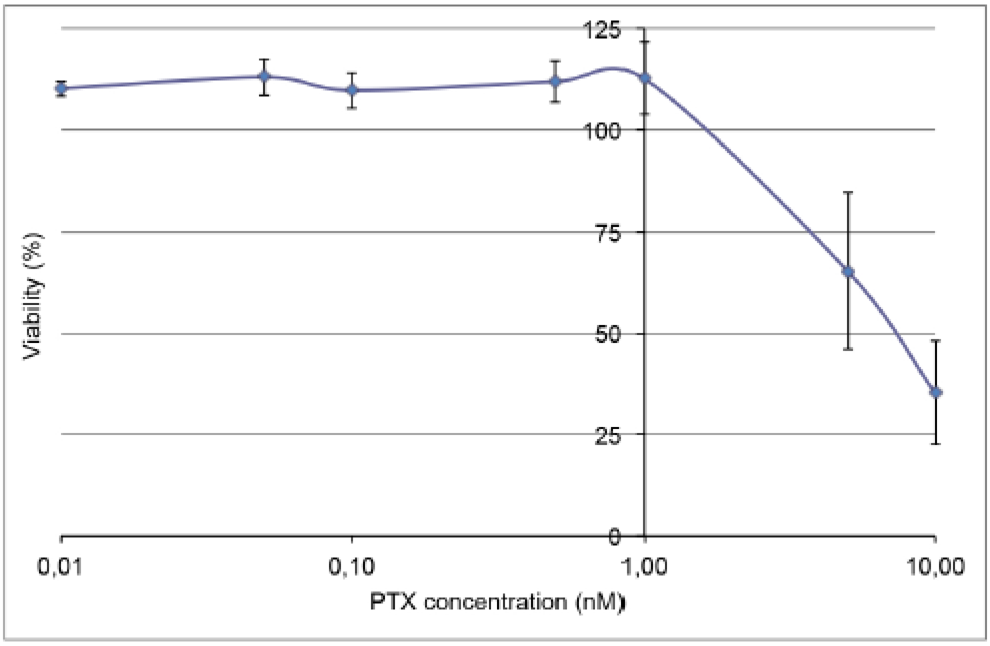

Figure S1. Effect of PTX on the viability of HeLa cells. Cells were incubated for 48 hours with increasing concentrations of PTX. The percentage of viable cells was calculated following a MTT assay. Data are presented as mean ± SD of three independent experiments.

A

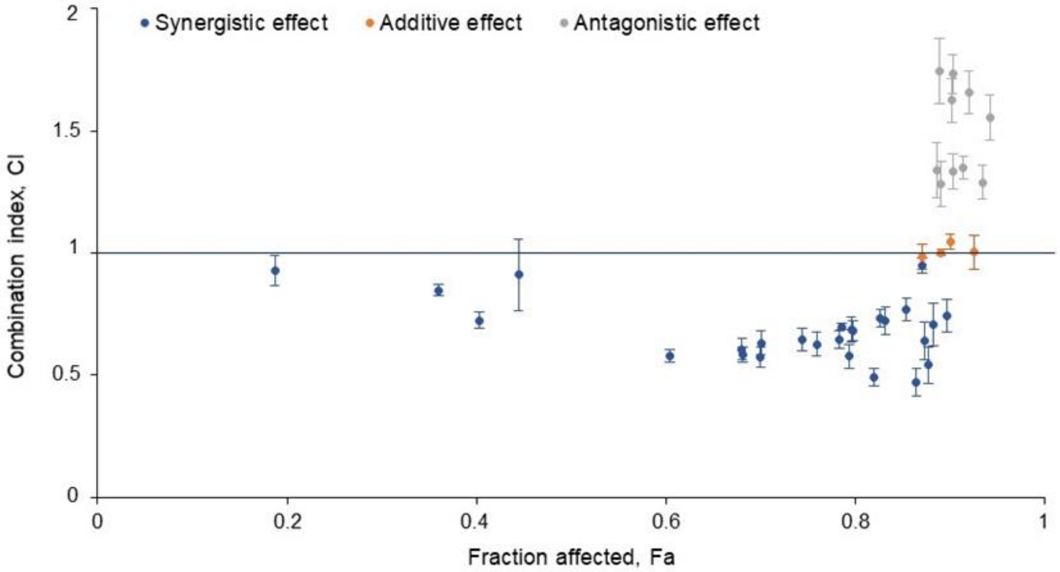

B

Synergistic effect

Carba1 (μM)	PTX (nM)	Fractional effect	CI
25	0.5	0.87	0.47 ± 0.06
25	0.25	0.82	0.49 ± 0.04
25	1	0.88	0.54 ± 0.07
12	1	0.70	0.57 ± 0.04
18	1	0.79	0.58 ± 0.05
3	1.5	0.60	0.58 ± 0.03
6	1.5	0.68	0.58 ± 0.03
18	0.5	0.68	0.61 ± 0.04
12	1.5	0.76	0.63 ± 0.05
3	2	0.70	0.63 ± 0.05
25	1.5	0.87	0.64 ± 0.08
6	2	0.74	0.65 ± 0.04
3	2.5	0.78	0.65 ± 0.03
18	1.5	0.80	0.68 ± 0.04
12	2	0.80	0.69 ± 0.05
6	2.5	0.79	0.70 ± 0.01
25	2	0.88	0.71 ± 0.09
18	2	0.83	0.72 ± 0.06
6	1	0.40	0.73 ± 0.03
12	2.5	0.83	0.73 ± 0.04
25	2.5	0.90	0.74 ± 0.07
18	2.5	0.86	0.77 ± 0.05
12	0.5	0.36	0.85 ± 0.02
18	0.25	0.45	0.91 ± 0.15
3	1	0.19	0.93 ± 0.06
3	5	0.87	0.95 ± 0.03

Additive effect

Carba1 (μM)	PTX (nM)	Fractional effect	CI
6	5	0.87	0.98 ± 0.05
12	5	0.89	1.00 ± 0.02
25	5	0.93	1.00 ± 0.07
18	5	0.90	1.05 ± 0.03

Antagonistic effect

Carba1 (μM)	PTX (nM)	Fractional effect	CI
3	7.5	0.89	1.28 ± 0.09
25	7.5	0.93	1.29 ± 0.07
12	7.5	0.90	1.33 ± 0.07
6	7.5	0.89	1.34 ± 0.11
18	7.5	0.92	1.35 ± 0.05
25	10	0.94	1.56 ± 0.09
3	10	0.90	1.62 ± 0.09
18	10	0.92	1.66 ± 0.09
12	10	0.90	1.73 ± 0.08
6	10	0.89	1.74 ± 0.13

Figure S2. Combination index (CI) values as a function of Fraction affected (Fa) in HeLa cells exposed to combination drug treatment. (**A**) The Fa-CI plot. Cells were incubated for 72 hours with the indicated combinations of Carba1/PTX. The percentage of viable cells was calculated following a Prestoblue assay. The Chou-Talalay method of synergy determination was used, along with CompuSyn software. Fa indicates the fractional inhibition for each combinational index (i.e., Fa = 0.5 corresponds to 50% of cell proliferation inhibition). The horizontal line at 1 indicates the line of additive effect. CI values less than, equal to, or greater than 1 indicate synergy, additivity, or antagonism, respectively. (**B**) Combination Index for each combination. CI were ranked in ascending order. The combination of Carba1 and PTX (12 μM/1 nM) is indicated in blue. Data are presented as mean ± SEM of 3 independent experiments.

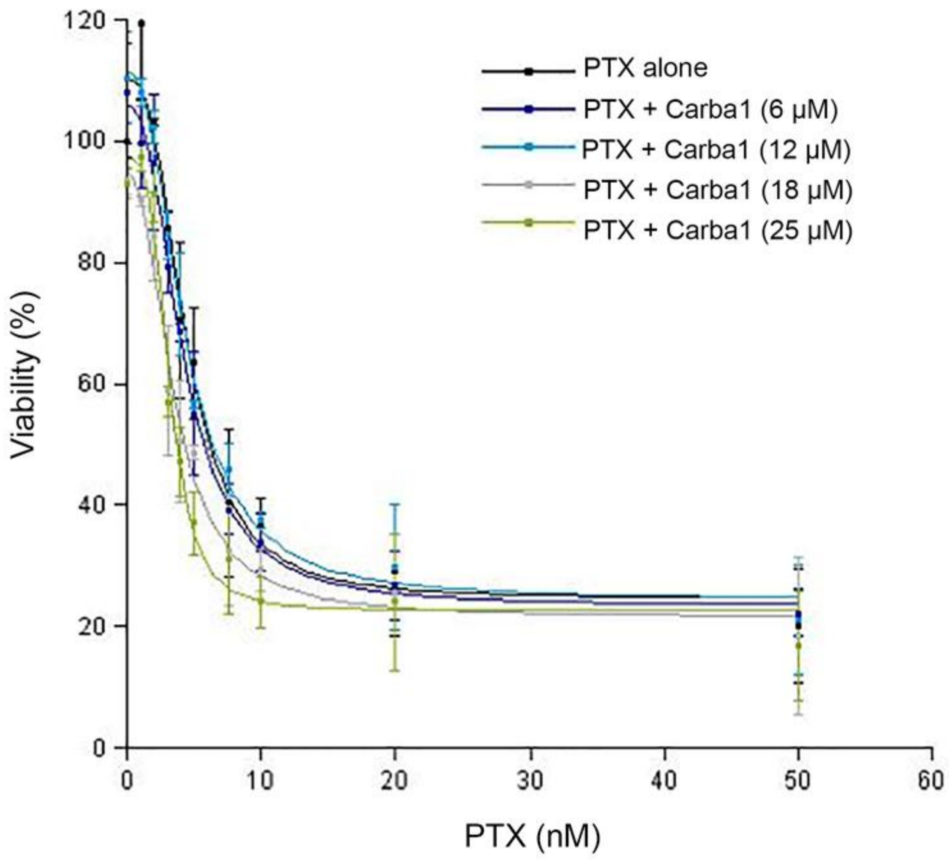

Figure S3. Effect of Carba1/PTX combination on the viability of RPE-1 cells. Cells were incubated for 72 hours with the indicated combinations of Carba1/PTX. The percentage of viable cells was calculated following a Prestoblue assay. Data are presented as mean ± SEM of 3 independent experiments. Note that the effect on cell viability of the different doses of Carba1 alone can be seen at the x-coordinate = 0, corresponding to 0 nM PTX.

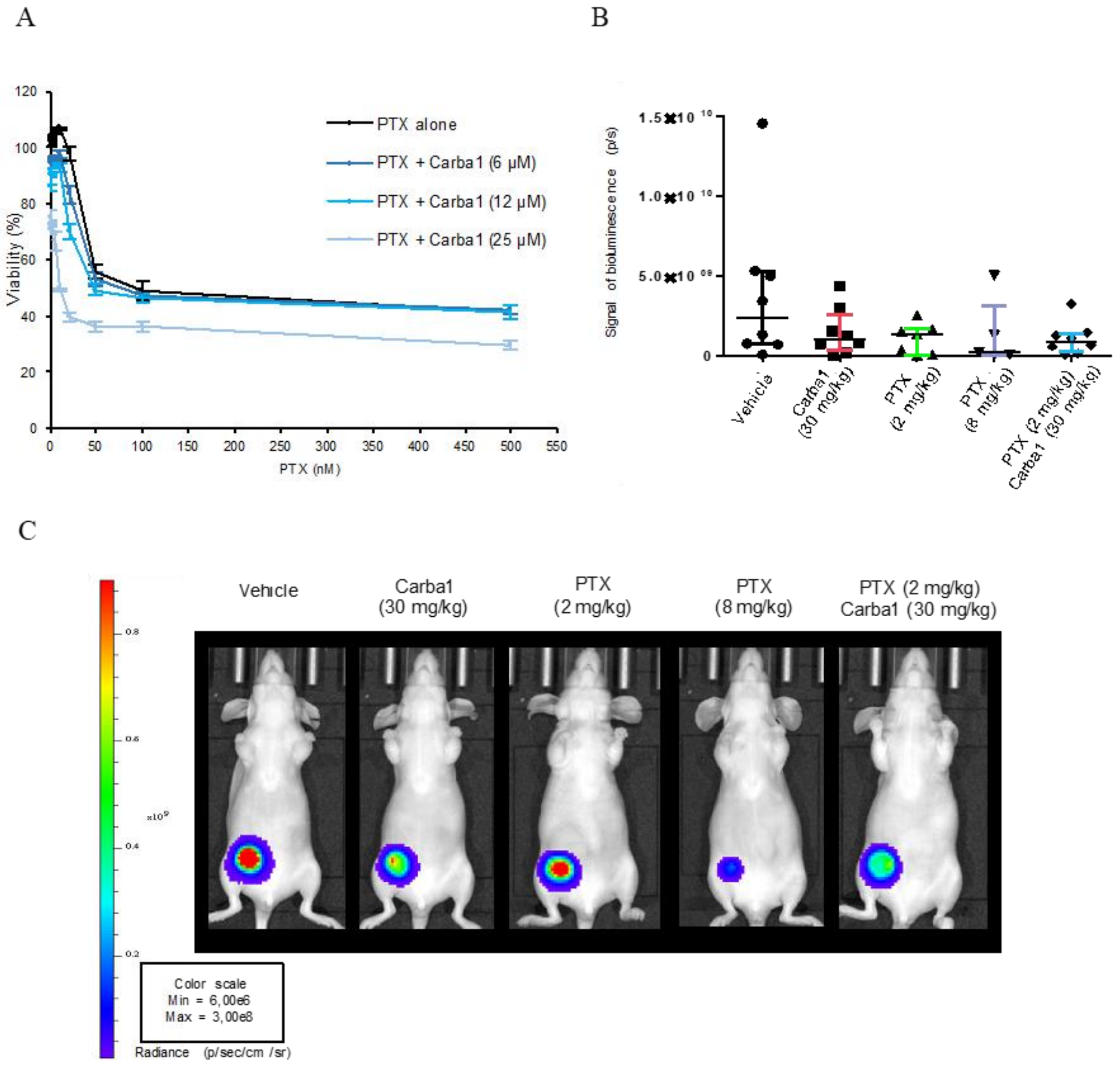

Figure S4. Effect of Carba1/PTX combination on 4T1 cells in vitro and in vivo. (**A**) Effect of Carba1/PTX combination on the viability of 4T1 cells. Cells were incubated for 72 hours with the indicated combinations of Carba1/PTX. The percentage of viable cells was calculated following a Prestoblue assay. Data are presented as mean ± SEM of 3 independent experiments. Note that the effect on cell viability of the different doses of Carba1 alone can be seen at the x-coordinate = 0, corresponding to 0 nM PTX, (**B**) Bioluminescence signals from orthotopic 4T1rvluc2 mammary tumors after 16 days of treatment by PTX, Carba1 and their combination. Error bars = SEM. n = 8 for vehicle, Carba1 alone, Carba1/PTX groups, n = 7 for PTX (2 mg/kg) group and n = 5 for PTX (8 mg/kg) group, (**C**) Bioluminescence images of orthotopic 4T1rvluc2 mammary tumor bearing mice after 16 days of treatment (one representative mouse per condition).

A

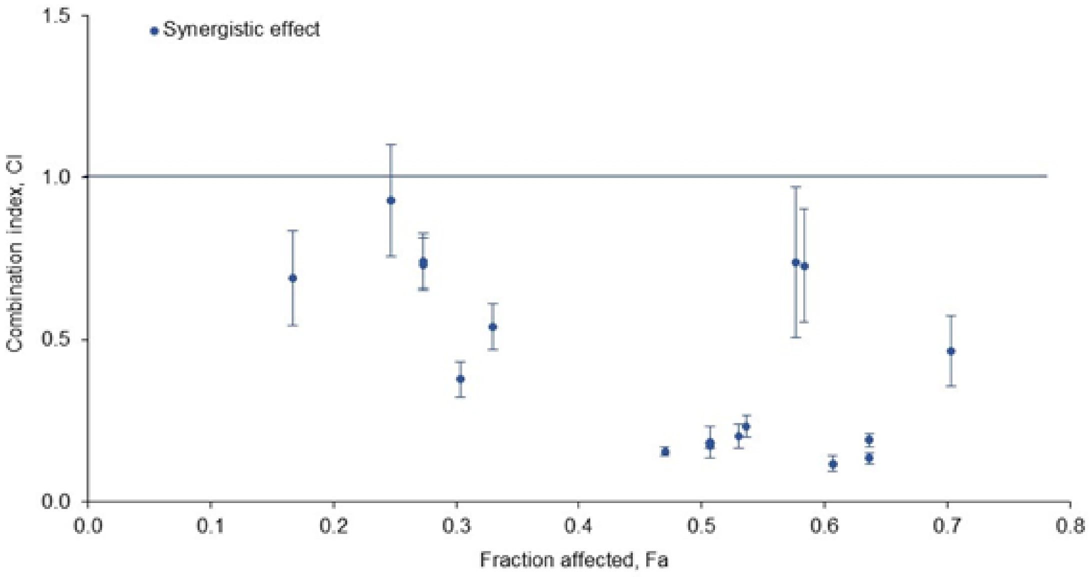

B

Synergistic effect

Carba1 (μM)	PTX (nM)	Fractional effect	CI
25	20	0.61	0.12 ± 0.02
25	50	0.64	0.13 ± 0.02
6	50	0.47	0.16 ± 0.01
12	50	0.51	0.17 ± 0.01
25	10	0.51	0.18 ± 0.05
25	100	0.64	0.19 ± 0.02
6	100	0.53	0.20 ± 0.04
12	100	0.54	0.23 ± 0.03
12	20	0.30	0.38 ± 0.06
25	500	0.70	0.46 ± 0.11
25	5	0.33	0.54 ± 0.07
6	20	0.17	0.69 ± 0.15
12	500	0.58	0.73 ± 0.17
25	0.5	0.27	0.73 ± 0.08
6	500	0.58	0.74 ± 0.23
25	0.1	0.27	0.74 ± 0.08
25	1	0.25	0.93 ± 0.17

Figure S5. Combination index (CI) values as a function of Fraction affected (Fa) in 4T1 cells exposed to combination drug treatment. (**A**) The Fa-CI plot. Cells were incubated for 72 hours with the indicated combinations of Carba1/PTX. The percentage of viable cells was calculated following a Prestoblue assay. The Chou-Talalay method of synergy determination was used, along with CompuSyn software. Fa indicates the fractional inhibition for each combinational index (i.e., Fa = 0.5 corresponds to 50% of cell proliferation inhibition). The horizontal line at 1 indicates the line of additive effect. CI values less than, equal to, or greater than 1 indicate synergy, additivity, or antagonism, respectively. (**B**) Combination Index for each combination. CI were ranked in ascending order. Data are presented as mean ± SEM of 3 independent experiments.

A

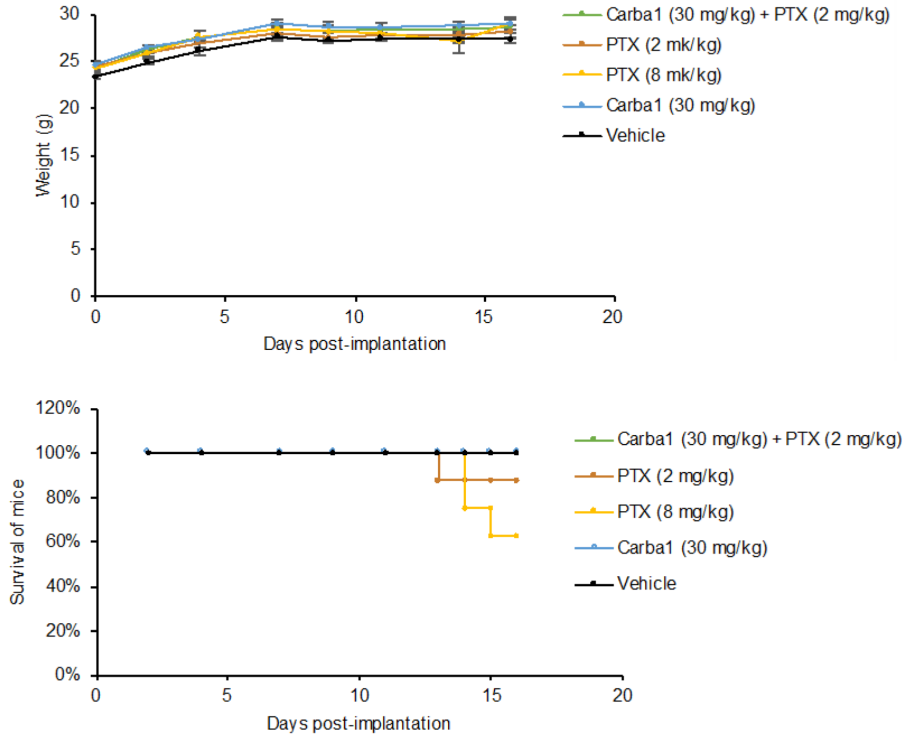

B

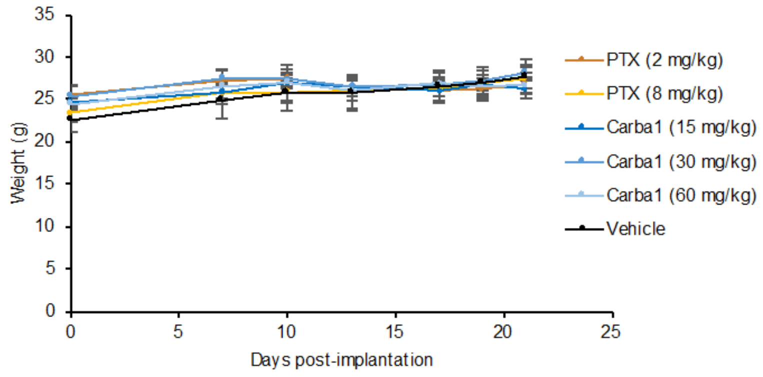

C

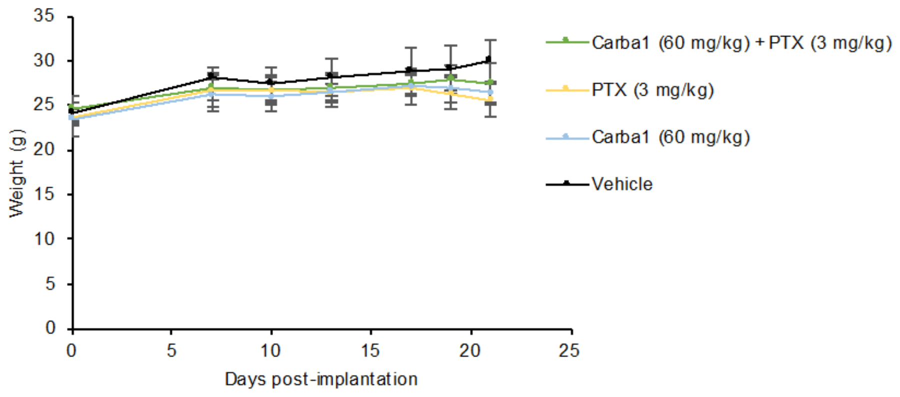

Figure S6. Weight and survival curves of mice treated with PTX, Carba1 and the Carba1/PTX combination. (**A**) Orthotopic 4T1 allografts model: weight and survival curves of mice treated with Carba1 (30 mg/kg), PTX (2 mg/kg), PTX (8 mg/kg) and the combination Carba1 (30 mg/kg)/PTX (2 mg/kg) (**B**) HeLa cells xenografts: weight of PTX and Carba1 treated mice (**C**) HeLa cells xenografts:

Weight of mice treated with Carba1 (60 mg/kg), PTX (3 mg/kg) and the combination Carba1 (60 mg/kg)/PTX (3 mg/kg).

Table S1. Analysis of the effect of PTX (1 nM) and Carba1 (25 µM) on microtubule dynamics in cells.

**Parameters**	**DMSO 0.25%**	**PTX 1 nM**	**Carba1 25 µM**
% time spent growing	84.19 ± 0.20	80.08 ± 0.25	60.40 ± 0.25***
% time spent in pause	15.81 ± 0.47	19.92 ± 0.50	39.60 ± 0.31***
Growth rate (µm/min ± SE)	14.65 ± 0.10	13.62 ± 0.14	10.20 ± 0.05***
Catastrophe frequency (μm^{-1} ± SE)	0.23 ± 0.03	0.27 ± 0,04	0.46 ± 0.05***
Catastrophe frequency (min^{-1} ± SE)	2.80 ± 0.61	2.81 ± 0,70	2.74 ± 0.70

HeLa cells were transfected with EB3-GFP, treated with or without indicated concentrations of drugs and followed by videomicroscopy. Data are from 3 independent experiments. For each experiment, 6 MTs/cell in 6 cells per condition were analyzed. *** $p < 0.001$, significantly different from control values (DMSO) using a Tukey test.

Table S2. Small molecule screening data.

**Category**	**Parameter**	**Description**
Assay	Type of assay	Cytotoxicity (MTT) assay on HeLa cells
	Primary measurement	Measurement of absorbance using a microplate reader
	Key reagents	HeLa cells, paclitaxel (PTX), DMSO
	Assay protocol	Seed cells (2500 cells/well). The day after, incubate cells for 48 hours with the compounds at a final concentration of 5 µM, with or without 1 nM PTX. Then, carry out MTT assay
	Additional comments	We observed that the composition of the serum in the cell culture medium could have an effect on the growth of the cells and on their response to the drugs. It is thus important to keep the same batch of serum throughout the screening and the subsequent analyses
Library	Library size	8000 molecules
	Library composition	Small, original and druggable molecules representative of the structural diversity of the CERMN's Chemical Library (> 19,000 compounds issued from numerous medicinal chemistry works).
	Source	Library from the Centre d'Etudes et de Recherche sur le Médicament de Normandie (part of the French CNRS chemical library, http://chimiotheque-nationale.cn.cnrs.fr)
	Additional comments	Compounds arrayed in 96-well plates as single compounds (5 mM) in DMSO
Screening Process	Format	96-well plates, clear, flat bottoms (Greiner)
	Concentration tested	5 µM, 0.1% DMSO
	Plate control	DMSO 0.1% was used as proliferation control and SDS 0.1% was used as a control of total cell death. These controls were included in each microplate, on the outer columns.
	Reagent/compound dispensing system	8-channel dispensers
	Detection instrument and software	FLUOstar OPTIMA microplate reader (BMG Labtechnologies)
	Normalization	Raw data converted into percentage of cell viability, based on plate control averages

Table S3. Carbazole-related compounds and their effect on HeLa cell viability.

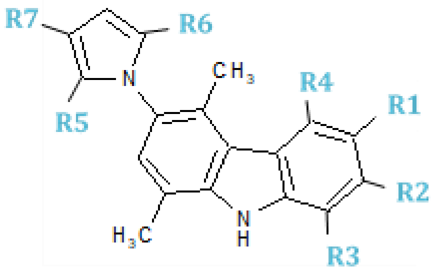

**Compound**			**Substituents at Position**					**HeLa Cells viability**
	R1	R2	R3	R4	R5	R6	R7	GI50 (μM)
Carba1	Cl	H	H	H	H	H	H	21.8 ± 1.2
T21	Br	H	H	H	H	H	H	16.2 ± 1.3
T22	Br	H	H	H	CH_3	CH_3	H	-
T23	OH	H	H	H	H	H	H	-
T25	F	H	H	H	CH_3	CH_3	H	-
T26	EtOCOO	H	H	H	CH_3	CH_3	H	-
T27	EtOCOO	H	H	H	H	H	H	-
T28	Cl	H	H	H	CH_3	CH_3	H	-
T29	F	H	H	H	H	H	CHO	14.2 ± 1.8
T30	Br	H	H	H	H	H	CHO	1.06 ± 0.04
T31	H	H	Et	H	H	H	H	-
T32	H	Cl	H	H	H	H	H	14.8 ± 1.6
T33	H	H	H	Cl	H	H	H	-

Table S4. Carba1 moderately inhibits the proliferation of cancer cells included in the NCI-60 panel.

Panel/Cell Line	Growth Percent
Leukemia	
CCRF-CEM	85.38
HL-60(TB)	133.20
K-562	49.55
MOLT-4	77.21
RPMI-8226	85.20
SR	52.83
Non-Small Cell Lung Cancer	
A549/ATCC	88.35
EKVX	92.80
HOP-62	90.90
HOP-92	100.86
NCI-H226	122.19
NCI-H23	98.27
NCI-H322M	92.42
NCI-H460	95.18
NCI-H522	62.95
Colon Cancer	
COLO 205	104.78
HCC-2998	107.35
HCT-116	92.42
HCT-15	75.26
HT29	91.81
KM12	79.44
SW-620	87.14
CNS Cancer	
SF-268	104.08
SF-295	99.71
SF-539	103.31
SNB-19	97.88
SNB-75	87.02
U251	94.87
Melanoma	
LOX IMVI	98.67
MALME-3M	106.66
M14	88.27
MDA-MB-435	78.54
SK-MEL-2	95.08
SK-MEL-28	96.41
SK-MEL-5	97.77
UACC-257	97.82
UACC-62	74.41
Ovarian Cancer	
IGROV1	96.13
OVCAR-3	99.28
OVCAR-4	90.68
OVCAR-5	101.61
OVCAR-8	98.92
NCI/ADR-RES	90.34
SK-OV-3	91.11
Renal Cancer	
786-0	96.62
A498	75.30
ACHN	95.42
CAKI-1	79.35
RXF 393	104.77
SN12C	86.04
TK-10	115.87
UO-31	88.88
Prostate Cancer	
PC-3	101.48
DU-145	90.74
Breast Cancer	
MCF7	84.88
MDA-MB-231/ATCC	100.33
HS 578T	87.42
BT-549	101.13
T-47D	63.68
MDA-MB-468	106.51
Mean	92.21
Delta	42.66
Range	83.65

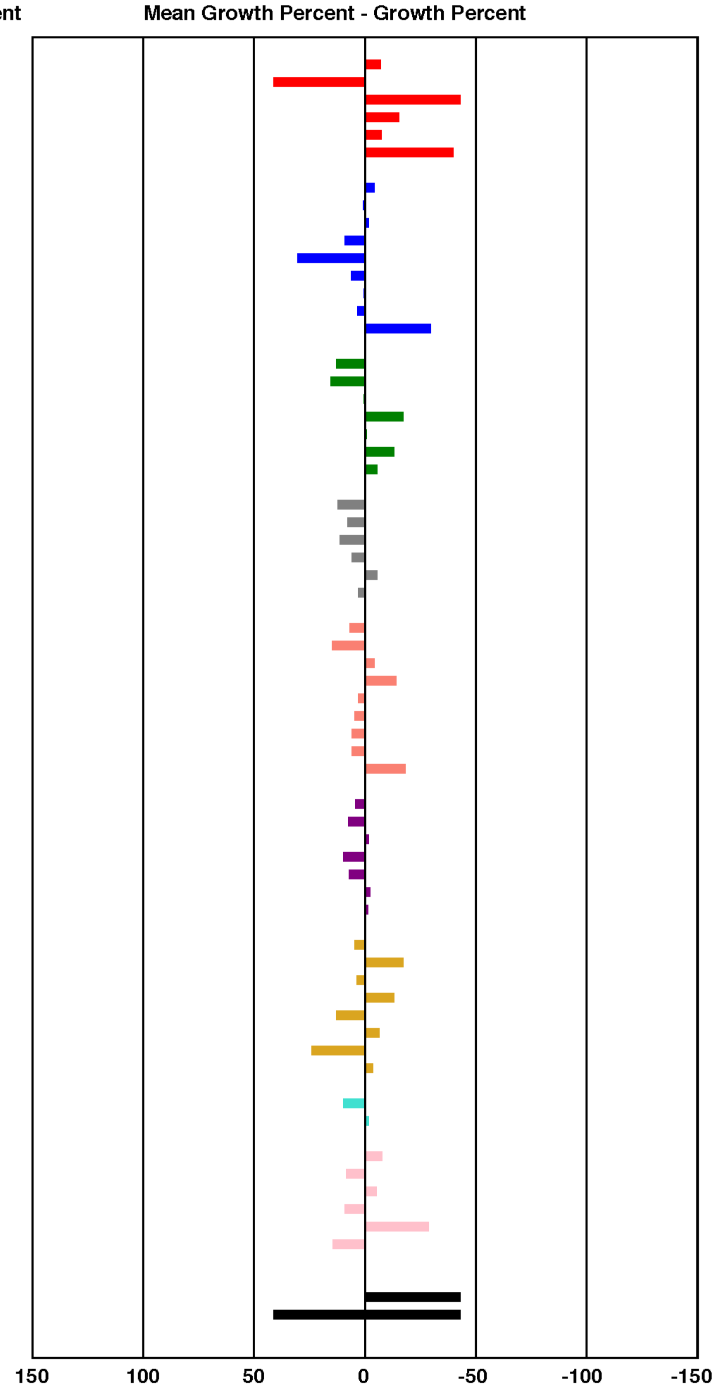

The drug's efficacy (Carba1, 10 µM) against the NCI-60 cancer cell lines was examined. The panel of cell lines analyzed included: six leukemia cell lines, nine non-small cell lung cancer cell lines, seven colorectal cancer cell lines, six CNS cancer cell lines, nine melanoma cell lines, seven ovarian cancer cell lines, seven renal cancer cell lines, two prostate cancer cell lines and six breast cancer cell lines. The screening was carried out by the US National Cancer Institute (NCI) using a sulforhodamine B (SRB) colorimetric assay. The data are reported as the percent growth of treated cells. The number reported is growth relative to the no-drug control, and relative to the time zero number of cells. This allows detection of both growth inhibition (values between 0 and 100) and lethality (values less than 0), as indicated by the color bar chart.

Table S5. Quantification of the fate of HeLa cells treated with Carba1 and/or PTX.

Duration of Observation	12 Hours						20 Hours
	Control	Carba1 (12 μM)	Carba1 (25 μM)	PTX (1 nM)	PTX (5 nM)	Carba1 (12 μM) + PTX (1 nM)	Carba1 (25 μM)
**Mitotic catastrophe**	0%	0%	The majority of cells stay blocked in pro-metaphase	0%	0%	5%	61%
**Aberrant mitosis**	3%	11%		25%	93%	83%	29%
**Normal mitosis**	97%	89%		75%	7%	12%	10%
	$n = 73$	$n = 68$		$n = 100$	$n = 43$	$n = 44$	$n = 105$

HeLa Kyoto cells were treated with the indicated concentrations of drugs and followed during 12 or 20 hours using time-lapse videomicroscopy. n represents the number of mitotic cells analyzed. Carba1 (12 μM) and PTX (1 nM) induced a slight increase of aberrant mitosis as compared to control (DMSO). At a higher concentration (25 μM), Carba1 induced cell prometaphase arrest followed by apoptotic death. PTX at 5 nM and the Carba1/PTX combination induced a mitotic delay followed by aberrant division.

Table S6. Profile of kinase inhibitory activity of Carba1.

Protein Kinase	Activity	Protein Kinase	Activity
Abl(h)	141	LIMK1(h)	117
ALK(h)	110	LKB1(h)	110
AMPKα1(h)	99	MAPK1(h)	106
AMPKα2(h)	104	MARK1(h)	102
ARK5(h)	87	Met(h)	86
Aurora-A(h)	105	MLCK(h)	82
Aurora-B(h)	72	MLK1(h)	83
BrSK1(h)	101	MRCKα(h)	140
BrSK2(h)	134	MRCKβ(h)	136
c-RAF(h)	100	mTOR(h)	98
CaMKI(h)	102	NEK2(h)	111
CaMKIIβ(h)	67	p70S6K(h)	110
CaMKIIγ(h)	104	PAK2(h)	103
CaMKIIδ(h)	74	PAR-1Bα(h)	99
CaMKIV(h)	85	PDGFRα(h)	112
CaMKIδ(h)	127	PDGFRβ(h)	126
CDK2/cyclinA(h)	110	PKA(h)	141
CDK7/cyclinH/MAT1(h)	115	PKBα(h)	116
cKit(h)	97	PKBβ(h)	116
cSRC(h)	100	PKCζ(h)	113
eEF-2K(h)	145	PKCι(h)	71
EGFR(h)	125	Plk1(h)	112
EphA2(h)	102	Plk3(h)	99
FAK(h)	104	Pyk2(h)	107
FGFR1(h)	72	Ret(h)	90
FGFR2(h)	94	ROCK-I(h)	107
FGFR3(h)	99	ROCK-II(h)	109
Flt3(h)	134	SIK(h)	124
GSK3β(h)	73	Src(1-530)(h)	100
IGF-1R(h), activated	92	Syk(h)	103
IR(h), activated	81	TAK1(h)	94
JAK2(h)	106	TGFBR1(h)	98

The kinase activity determined in the presence of 10 μM Carba1 is expressed as percentage of the control activity without inhibitor. Final concentration of ATP in the experiment was 100 μM.

Movie S1. Carba1 induces a delay in mitosis.

Time-lapse microscopy was performed during 12 hours, on HeLa Kyoto cells treated with DMSO (control), 12 µM or 25 µM Carba1. Images were acquired every 2.5 minutes. Bar = 10 µm.

Movie S2. Carba1 blocks cells in mitosis and induces mitotic catastrophe.

Time-lapse microscopy was performed during 20 hours, on HeLa Kyoto cells treated with Carba1 (25 µM). Images were acquired every 2.5 minutes. Bar = 10 µm.

Movie S3. Effects of PTX (1 nM and 5 nM) and of the combination of Carba1 and PTX (12 µM/1 nM) on HeLa Kyoto cell mitosis.

Time-lapse microscopy was performed, during 12 hours on HeLa Kyoto cells treated with 1 nM or 5 nM PTX and the Carba1 PTX (12 µM/1 nM) combination, as indicated. Images were acquired every 2.5 minutes. Bar = 10 µm.

© 2020 by the authors. Licensee MDPI, Basel, Switzerland. This article is an open access article distributed under the terms and conditions of the Creative Commons Attribution (CC BY) license (http://creativecommons.org/licenses/by/4.0/).
